# NiGA MOF-based dispersive micro solid phase extraction coupled to temperature-assisted evaporation using low boiling point solvents for the extraction and preconcentration of butylated hydroxytoluene and some phthalate and adipate esters

**DOI:** 10.1039/d3ra04612e

**Published:** 2023-10-17

**Authors:** Sakha Pezhhanfar, Mir Ali Farajzadeh, Seyed Abolfazl Hosseini-Yazdi, Mohammad Reza Afshar Mogaddam

**Affiliations:** a Department of Analytical Chemistry, Faculty of Chemistry, University of Tabriz Tabriz Iran mafarajzadeh@yahoo.com mafarajzadeh@tabrizu.ac.ir +98 41 33340191 +98 41 33393084; b Engineering Faculty, Near East University 99138 Nicosia, North Cyprus Mersin 10 Turkey; c Department of Inorganic Chemistry, Faculty of Chemistry, University of Tabriz Tabriz Iran; d Food and Drug Safety Research Center, Tabriz University of Medical Sciences Tabriz Iran; e Pharmaceutical Analysis Research Center, Tabriz University of Medical Sciences Tabriz Iran

## Abstract

The first-ever attempt to apply nickel gallic acid metal–organic framework (NiGA MOF) in analytical method development was done in this research by the extraction of some plasticizers from aqueous media. The greenness of the method is owing to the use of gallic acid and nickel as safe reagents and water as the safest solvent. Low boiling point solvents were applied as desorption solvents that underwent temperature-assisted evaporation in the preconcentration step. Performing the evaporation using a low-temperature water bath for a short period of time streamlines the preconcentration section. Into the solution of interest enriched with sodium sulfate, a mg amount of NiGA MOF was added alongside vortexing to extract the analytes. Following centrifugation and discarding the supernatant, a μL level of diethyl ether was added onto the analyte-loaded NiGA MOF particles and vortexed. The analyte-enriched diethyl ether phase was transferred into a conical bottom glass test tube and located in a water bath set at the temperature of 35 °C under a laboratory hood. After the evaporation, a μL level of 1,2-dibromoethane was added to the test tube and vortexed to dissolve the analytes from the inner perimeter of the tube. One microliter of the organic phase was injected into a gas chromatograph equipped with flame ionization detection. Appreciable extraction recoveries (61–98%), high enrichment factors (305–490), low limits of detection (0.80–1.74 μg L^−1^) and quantification (2.64–5.74 μg L^−1^), and wide linear ranges (5.74–1000 μg L^−1^) were obtained at the optimum conditions.

## Abstract 2

The first-ever attempt to apply nickel gallic acid metal–organic framework (NiGA MOF) in analytical method development was done in this research by the extraction of some plasticizers from aqueous media.

## Graphical abstract

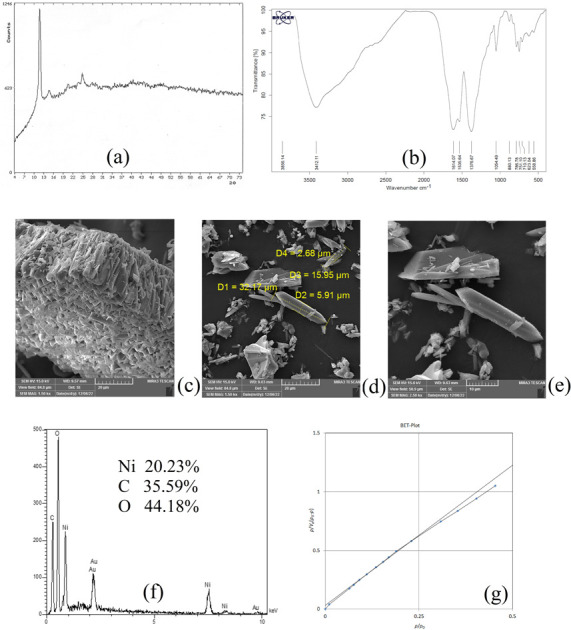

## 1. Introduction

Adipate and phthalate esters are categorized as plasticizers that are exploited to increase the flexibility of plastic containers used for food and drink packaging.^1^ Although they act successfully to soften plastic bottles and containers, their entrance into the content of the containers due to low molecular weights and not having chemical bonds with the polymers^2,3^ such as polyethylene terephthalate (PET)^4^ and polyvinyl Chloride (PVC)^5^ is a big concern. Subsequently, the entrance of plasticizers into the human body is health-threatening. They have been detected even in amniotic fluid, breast milk, and serum.^6^ The maximum contaminant levels of di(2-ethylhexyl)adipate (DEHA) and di(2-ethylhexyl)phthalate (DEHP) have been documented to be 400 and 6 μg L^−1^, respectively.^7^ DEHA is also associated with liver cancer in mice^6^ and postnatal death in rats.^8^ DEHP which is used in PVC medical packages such as blood bags is known to be carcinogenic for humans. Its tolerable daily intake is 50 μg kg^−1^ per body weight per day.^9^ DEHP has also shown DNA damage to human lymphocytes.^10^ Di-*iso*-butyl phthalate (DIBP) triggers male and female reproductive toxicity. It also results in adverse effects on the liver. Moreover, DIBP presence in the body is associated with the risk of diabetes.^11^ Di-*n*-butyl phthalate (DNBP) decreases progesterone production at mid-pregnancy.^12^ DNBP and DIBP have shown genotoxicity in human epithelial cells of the upper aerodigestive tract.^13^ DNBP was documented to be correlated with DNA damage to human mucosal cells and DIBP is linked with lymphocytes' DNA damage.^14^ Also, to restrict the oxidation of polymeric compounds, butylated hydroxytoluene (BHT), as an antioxidant, is added to polymers.^15^ The maximum limit of BHT, butylated hydroxyanisole (BHA), and *tert*-butyl hydroquinone (TBHQ) should not be more than 200 mg kg^−1^ (either single or in combination) in oil samples.^16^ BHT was observed to be toxic to the neurobehavioral activity of rats. Also, it shows pathological effects on the brain, heart, and lungs.^17^

According to the health-threatening effects of the compounds of interest, they should be monitored in foods and beverages stored in plastic containers. Up to now, high-performance liquid chromatography^18^ and gas chromatography (GC)^19^ have been used for monitoring BHT, and phthalate and adipate esters. Since direct analysis is rarely possible using GC or results in high limits of detection (LODs) and quantification (LOQs) and also suffers from the matrix effect of the real samples, sample preparation procedures are inevitably necessary to be applied on samples prior to their injection into analytical apparatuses. Solid phase extraction,^20^ liquid–liquid extraction,^21^ solid phase microextraction,^22^ headspace solid phase microextraction,^23^ and dispersive liquid–liquid microextraction (DLLME)^24^ have been performed for the extraction of the target compounds. The evolved version of dispersive solid phase extraction was introduced as dispersive micro solid phase extraction (DμSPE) by applying low sorbent weights which makes the approach more efficient.^25^ Although DμSPE is beneficial, a preconcentration method is needed to couple with it in order to dwindle the LOD and LOQ values. Previously, DLLME has been coupled to DμSPE.^26^ To ease the extraction process, this study eliminates the use of DLLME and applies temperature-assisted evaporation (TAE) by using low boiling point desorption solvents for the preconcentration aim.

Metal–organic frameworks (MOFs) as hybrid and crystalline coordination polymers have revolutionized various fields including sample preparation,^27^ supercapacitors,^28^ and water treatment.^29,30^ Specifically in the field of extraction, MOF-70,^31^ MIL-101(Cr),^32^ MIL-68 (Al),^33^ ZIF-8,^34^ MIL-53 (Cr),^35^ Basolite F300 MOF,^36^ magnetic graphene@ZIF-8,^37^ TMU-23@TMU-24,^38^ and TMU-6 (ref. 39) have been utilized for sample preparation of matrices containing plasticizers. The application of bio-MOFs is missing among MOF uses for the extraction of plasticizers. Bio-MOFs are superior to MOFs due to being synthesized from biologically active compounds, green, medium-compatible, nontoxic, and well-dispersed in solutions.^40,41^

Because of the plasticizers' addition to the structure of polymers, they can enter into different liquids that are stored in plastic bottles. Since plasticizers have health-threatening effects, their presence in different edible stuff has to be monitored. It is worth mentioning that their direct analysis in samples is barely possible due to the matrix effect of samples and their low concentrations. So, they have to be extracted, preconcentrated, and subsequently injected into analytical instruments. Plastic bottled water samples were selected to monitor the quality of the stored drinkable water in this study. Moreover, their presence in tap water was investigated due to the spread of plastic pipes in the construction industry. Furthermore, based on the widespread utilization of plasticizers and the environment's contamination, their presence was also monitored in rainwater samples. For the first time in this study, a bio-MOF called nickel-gallic acid MOF (NiGA MOF) was applied for the extraction of some phthalate and adipate esters and BHT. Using a bio-MOF instead of an MOF is an asset for the study. Applying no organic solvents for the sorbent preparation is also an asset. The approach is green owing to the use of nickel, gallic acid, and water in the synthesis process. No long reaction time, high reaction temperature, and expensive apparatus are needed to propel the bio-MOF synthesis. The low weight of bio-MOF used in the extraction process is also appreciable. The elimination of DLLME streamlined the procedure by reducing the applied tools, organic solvent volumes, and the analyst's fatigue. Centrifugation was also eliminated from the preconcentration step. The reasons for the selection of NiGA MOF in this study can be summarized as being composed of green reagents (nickel and gallic acid), application of the safest solvent (water), and no need for high temperatures in the synthesis process, being bio-MOF and benefiting from its related natural advantages, medium compatibility, well dispersion into the aqueous medium, and the ability for the creation of intermolecular bonds with the surveyed analytes (see Section 3.6.). Based on the given facts, both the extraction and preconcentration steps are economical which is precious. Also, for the first time, NiGA MOF-based DμSPE was coupled to TAE of the desorption solvent. A μL level of an organic solvent was applied to dissolve the residues obtained from the evaporation step and one microliter of it was injected into GC-flame ionization detection (FID).

## 2. Materials and methods

### 2.1. Chemicals and solutions

The utilized chemicals for the provision of NiGA MOF including nickel(ii) chloride hexahydrate (NiCl_2_·6H_2_O), gallic acid, and potassium hydroxide were provided by Merck (Darmstadt, Germany). Deionized water was bought from Ghazi Co. (Tabriz, Iran). The target compounds of the survey including BHT, DNBP, DIBP, DEHP, and DEHA were purchased from Sigma-Aldrich (St Louis, MO, the USA). Their chemical structures and physicochemical properties are consolidated in Table 1. The desorption solvents including diethyl ether (DE), *tert*-butyl methyl ether (TBME), carbon disulfide, *n*-pentane, and petroleum ether (PE) were provided by Sigma-Aldrich. Sodium chloride and sodium sulfate for performing the salting-out effect were from Merck. The elution solvents including carbon tetrachloride, 1,2-dibromoethane (1,2-DBE), and 1,1,1-trichloroethane (1,1,1-TCE) were purchased from Janssen (Beerse, Belgium). Sodium hydroxide and hydrochloric acid solution (37%, w/w) were purchased from Merck and utilized for pH adjustment. A methanolic stock solution with a concentration of 250 mg L^−1^ (with respect to each analyte) was prepared and used for direct injection into the separation system and also spiking into the deionized water and surveyed aqueous samples.

**Table 1:** Chemical structures and physicochemical properties of the surveyed target compounds

Analyte	Structure	Molecular formula	Molecular weight (g mol^−1^)	Boiling point (°C)	Solubility in water at 25 °C (mg L^−1^)	Density (g mL^−1^)
BHT	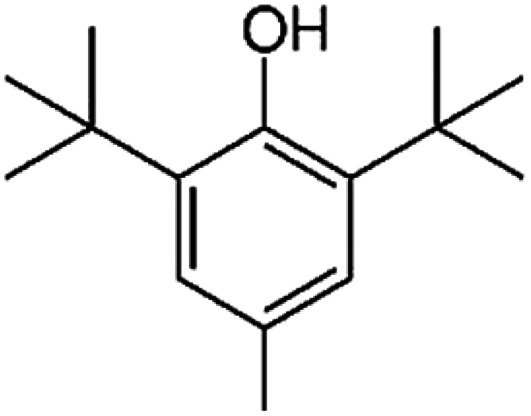	C_15_H_24_O	220.35	265	1.10	1.05
DIBP	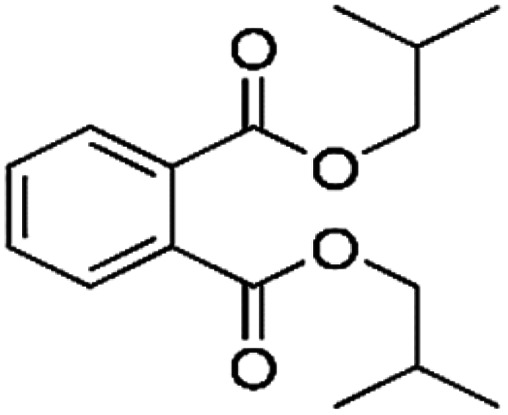	C_16_H_22_O_4_	278.35	320	6.20	1.04
DNBP	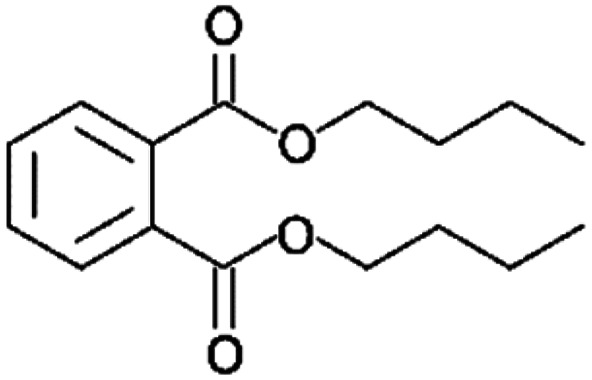	C_16_H_22_O_4_	278.34	340	11.20	1.05
DEHA	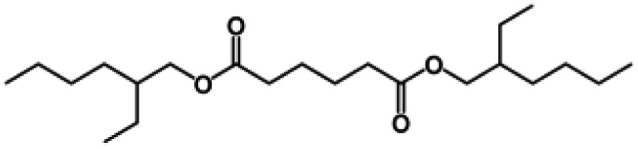	C_22_H_42_O_4_	370.60	416	0.78	0.92
DEHP	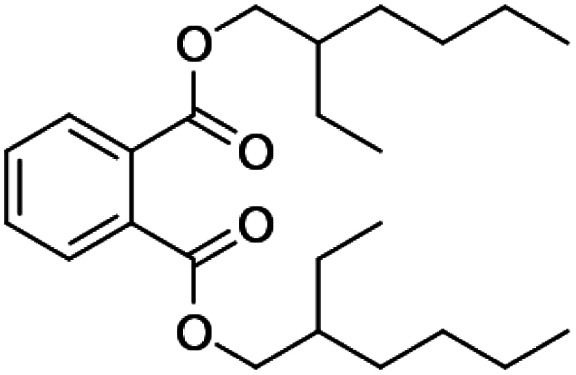	C_24_H_38_O_4_	390.60	385	0.27	0.98

### 2.2. Samples

Four freshly-produced bottled water samples were bought from a local hypermarket in Tabriz city (East Azerbaijan Province, Iran). They underwent the extraction and preconcentration method as were bought. Also, two tap water and two rainwater samples were collected from Tabriz city and subjected to the developed method. The samples were directly extracted with no dilution.

### 2.3. Apparatus

The separation of the five surveyed analytes was done using a Shimadzu gas chromatograph (2014, Kyoto, Japan) with an FID and a splitless/split injection port. The temperature of the column oven was fixed at 60 °C for 1 min and then increased to 300 °C at the rate of 18 °C min^−1^. It was maintained at 300 °C for 1 min finally. Zebron capillary column (5% diphenyl, 95% dimethyl polysiloxane; Phenomenex, Torrance, CA, the USA), (30 m × 0.25 mm i.d., with a film thickness of 0.25 μm) was used in the study. Helium (99.999%; Gulf Cryo, Dubai, United Arab Emirates) was used as the makeup (flow rate, 30 mL min^−1^) and carrier (linear velocity, 30 cm s^−1^) gasses. 300 °C was fixed for both FID and injection port. The sampling time and split ratio of the injection port were 1 min and 1 : 10, respectively. The air inlet of FID was set at 300 mL min^−1^ and the fuel (hydrogen) at the flow rate of 30 mL min^−1^ was generated by a Shimadzu hydrogen generator (OPGU-1500S). A Metrohm pH meter (Herisau, Switzerland), model 654, was utilized in the preparation of the samples. A Hettich centrifuge (D-7200, Kirchlengern, Germany) was used in the DμSPE step. A Falc (Labsonic LBS2) thermostatic and ultrasonic water bath (Treviglio, Italy) was used in the preconcentration step. For the dispersion of NiGA MOF into the solutions in order to facilitate the adsorption process, an L46 vortex (Labinco, Breda, the Netherlands) was used. A UT 12 Heraeus oven (Hanau, Germany) was applied to propel the synthesis of the bio-MOF. Different analyses including Brunauer–Emmett–Teller (BET, BELSORP-mini II, Japan) for surface area, total pore volume, and average pore diameter, scanning electron microscopy (SEM) (Mira 3 microscope, Tescan, Czech Republic) for the morphology of the bio-MOF, energy dispersive X-ray (EDX) for the elemental analysis, Fourier transform infrared (FTIR) spectrophotometry (Bruker, Billerica, USA) for the functional groups, and X-ray diffraction (XRD) (Siemens D500 diffractometer, and Siemens AG, Karlsruhe, Germany) for crystallinity evaluations were carried out on the synthesis product.

### 2.4. Synthesis of NiGA MOF

Based on upscaling the previously-introduced method,^42^ NiGA MOF was synthesized and used in the analytical method. Initially, 50 mL of 0.16 mol L^−1^ potassium hydroxide aqueous solution was prepared, and 10 mmol (2.38 g) NiCl_2_·6H_2_O and 20 mmol gallic acid (3.75 g) were added and sonicated for 30 min. The mixture was then transferred into a Teflon-lined stainless steel autoclave and heated for 24 h at the temperature of 120 °C. After the reaction was completed, the brown product was filtered and washed with 50 mL of deionized water. Then, it was dried at room temperature and transferred into a beaker and put in an oven for 24 h at the temperature of 100 °C for activation. Finally, the bio-MOF was collected and stored in a sealed airtight vial.

### 2.5. Extraction procedure

#### 2.5.1. DμSPE section

A 250 μg L^−1^ concentration of each target compound was spiked in 5 mL of deionized water located in a 10 mL conical bottom glass test tube. 750 mg of sodium sulfate (15%, w/v) was dissolved in the above-mentioned solution *via* vortexing to perform the salting-out effect. 15 mg of NiGA MOF was added into the solution of interest and vortexed for 5 min to streamline the adsorption of the analytes onto the bio-MOF particles. Following the process, 5 min centrifugation at the rate of 5000 rpm isolated the analyte-loaded NiGA MOF particles from the solution. 700 μL of DE was added onto the bio-MOF and the glass test tube was sealed using a lid and sealing film. Vortexing for 3 min was implemented to desorb the analytes from the NiGA MOF particles.

#### 2.5.2. TAE section

The analyte-enriched DE phase obtained from the above-mentioned section was poured into a conical bottom glass test tube and located in a thermostatic water bath set at the temperature of 35 °C under a laboratory hood. The DE phase evaporated using the set temperature. 10 μL of 1,2-DBE was added into the tube and vortexed for 3 min to dissolve the residues from the inner perimeter of the tube. One microliter of the organic phase was injected into the GC-FID system for analysis.

### 2.6. Enrichment factor and extraction recovery calculations

The performed preconcentration on the analytes through the method is shown by enrichment factor (EF). This term illustrates the ratio of the organic phase analyte concentration (*C*_org_) to the analyte's concentration in the aqueous phase (*C*_0_). Eqn (1) shows EF calculation.1
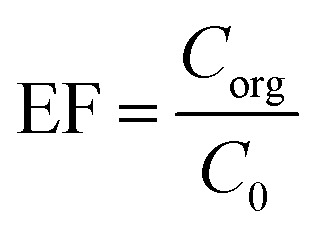


The ratio of the migrated analytes into the extracted phase is shown by extraction recovery (ER). Based on eqn (2) it is understood that the percentage of the migrated analyte number into the organic phase (*n*_fin_) to the same term in the aqueous solution (*n*_0_) is called ER.2
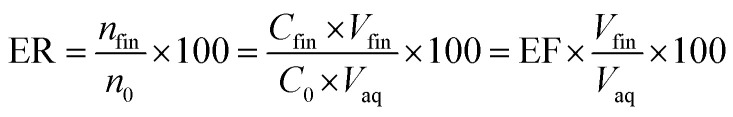


In this equation, *V*_aq_ is the volume of the initial aqueous phase and *V*_fin_ is the volume of the organic phase.

## 3. Results and discussion

### 3.1. Characterization of NiGA MOF

Once the desired bio-MOF was synthesized, XRD, SEM, BET, FTIR, and EDX analyses were carried out to reveal the chemical characteristics of the sorbent used in the method.

**Fig. 1:**
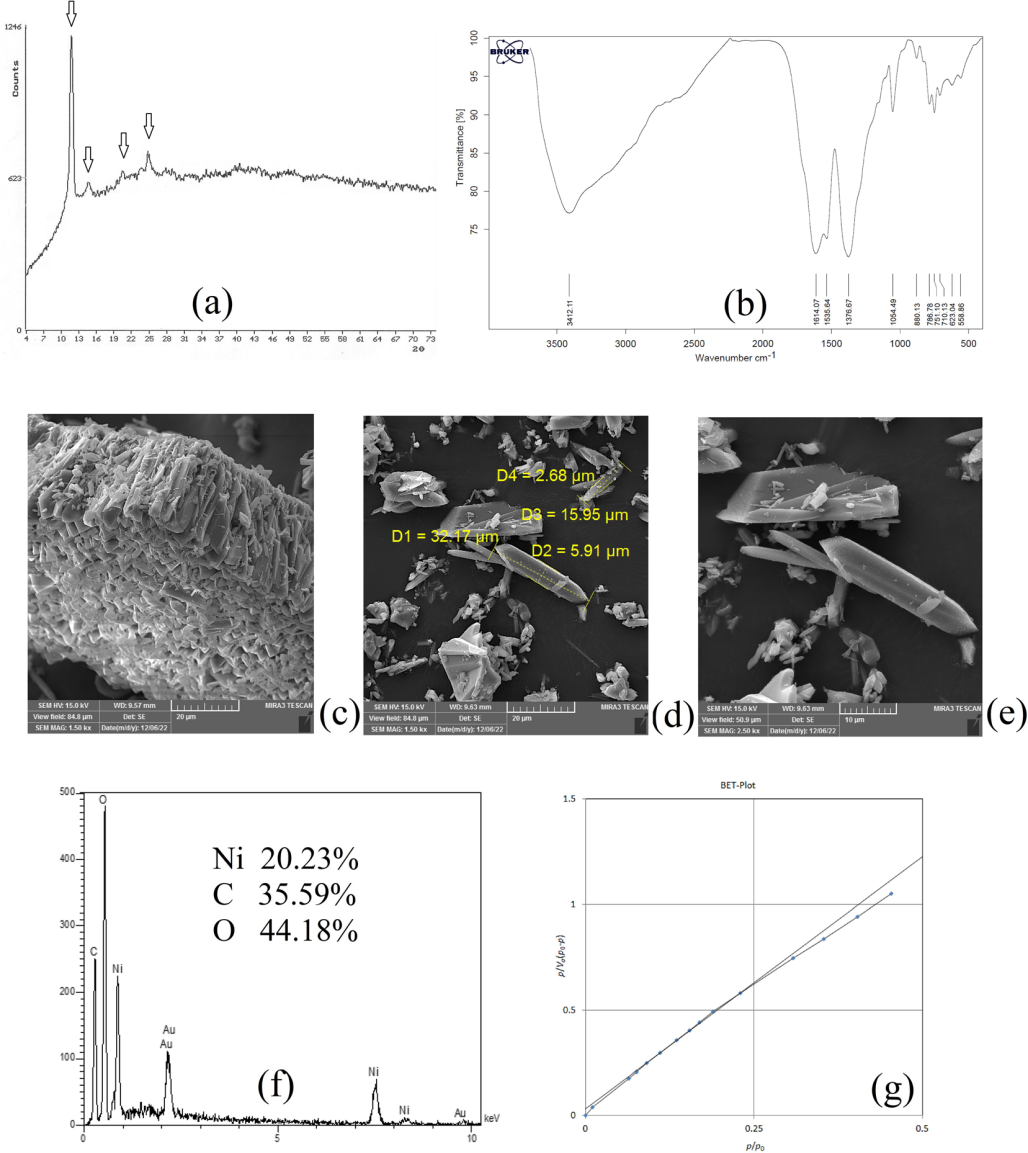
XRD pattern (a), FTIR spectrum (b), SEM images (c–e), EDX spectrum (f), and BET curve (g) of NiGA MOF.

XRD analysis was carried out on NiGA MOF to result in the XRD pattern of the coordination polymer. This pattern demonstrates the crystalline feature of the product. Fig. 1a shows the XRD pattern of NiGA MOF. The pattern is recorded at the 2*θ* range of 4–74°. As can be seen, there are some small XRD peaks in this window denoting the presence of different crystallographic planes in the structure of NiGA MOF. The planes are shown at *2θ* values of around 11, 14, 20, 21, 24, 25, 27, 36, and 41°. The low intensity of the crystallographic peaks and the upshift of the XRD pattern result in small XRD peaks. Moreover to the existence of various peaks showing the crystallographic planes of the bio-MOF, the obtained pattern's overlapping with a documented XRD pattern in the previous study proves the successful synthesis of NiGA MOF.^43^

FTIR analysis reveals the existence of different functional groups. The presence of functional groups streamlines the adsorption of target compounds onto the bio-MOF structure. Fig. 1b demonstrates the FTIR spectrum of NiGA MOF. The FTIR spectrum is recorded in the range of 400–4000 cm^−1^. The absorption peaks at 1614.07 and 1535.64 cm^−1^ are related to C=C stretching which stems from the cyclic section of the bio-MOF. The absorption peak at 1376.67 cm^−1^ ascribes C–H bending of the organic section of the framework. The 1054.49 cm^−1^ absorption peak shows C–O stretching which is the basis of bio-MOF formation through the deprotonation of the hydroxide groups that leads to oxygen–nickel bond creation. C=C bending triggered by the organic section of NiGA MOF is shown by 880.13, 786.78, 751.10, and 710.13 cm^−1^ absorption peaks. The observed peaks at 623.04 and 558.86 cm^−1^ prove the formation of nickel–oxygen bonds that paves the way to obtain NiGA MOF.

SEM analysis can be helpful by providing some informative data about the chemical's morphology, dimensions, and shape distributions. Fig. 1c–e illustrate the SEM images obtained by the implementation of a 15 000 V electron beam and work distances of 9.57, 9.63, and 9.63 mm, respectively. 1500, 1500, and 2500 times magnification scales were applied to get the illustrated SEM images, respectively. Fig. 1c reveals a μm-level layer of NiGA MOF resulting from vertical stacking of the bio-MOF particles creating a rugged surface. In Fig. 1d, it is seen that the longitudinal dimension of the bio-MOF particles is ranged from 15.95–32.17 μm. Also, the transverse dimension is ranged from 2.68–5.91 μm. Fig. 1e also demonstrates the needle-like morphology of the synthesized NiGA MOF.

EDX analysis provides surface elemental analysis of an MOF. By paying heed to the obtained results of EDX, the composing elements of an MOF are revealed and also the presence of any impurity or undesired element can be detected according to the analysis. Moreover, the percentage of the ligand elements and cation can be disclosed. The results of the EDX analysis carried out on NiGA MOF are shown in Fig. 1f. No extra peak except for the composing elements (nickel, carbon, and oxygen) are detected. The gold peak results from the applied procedure for gold coating of the sample. It is obtained that the surface of NiGA MOF is composed of 35.59% carbon, 44.18% oxygen, and 20.23% nickel.

BET analysis based on the adsorption and desorption of nitrogen gas is able to reveal average pore diameter, surface area, and total pore volume. Fig. 1g illustrates the obtained BET plot for the synthesized NiGA MOF. 19.39 nm average pore diameter, 0.0087 cm^3^ g^−1^ total pore volume, and 1.80 m^2^ g^−1^ surface area were the recorded data for the synthesized bio-MOF.

### 3.2. Optimization of effective parameters

#### 3.2.1. Optimization of the weight of NiGA MOF

**Fig. 2:**
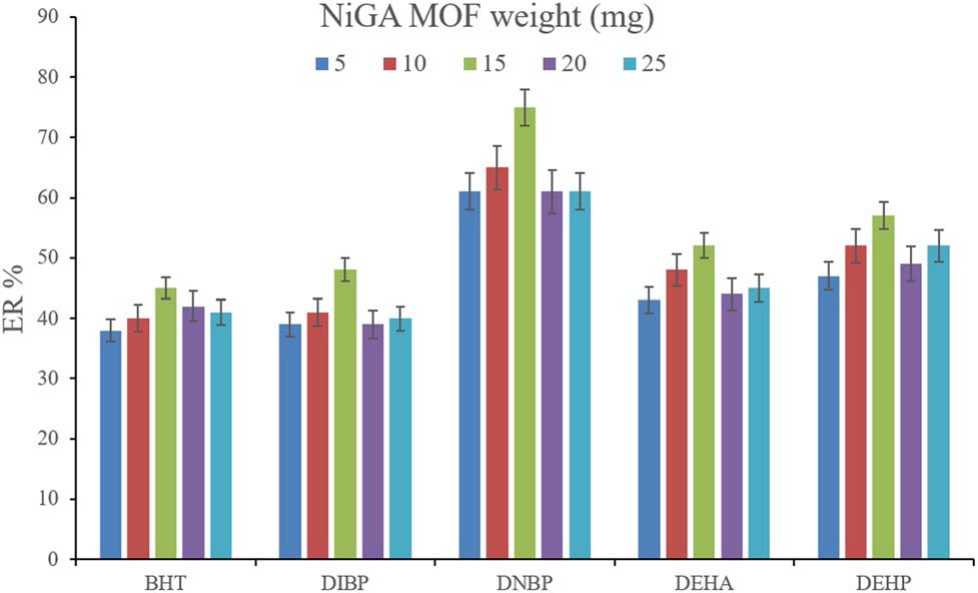
Optimization of NiGA MOF weight. Extraction conditions: DμSPE procedure: aqueous solution volume, 5 mL deionized water spiked with 250 μg L^−1^ of each analyte having 0.750 g dissolved Na_2_SO_4_; vortex time in adsorption step, 5 min; desorption solvent (volume), DE (500 μL); vortex time in desorption step, 5 min; and centrifugation speed and time, 6000 rpm and 5 min, respectively. TAE procedure: water bath temperature, 35 °C; solvent (volume), 1,2-DBE (10 μL); and vortexing time, 3 min. The error bars show the minimum and maximum of three repeated determinations.

In DμSPE-oriented procedures, the weight of sorbent is of great importance since it determines the adsorptive efficiency of the sorbent and the method's economical aspect. In the case of MOFs, this point is highlighted. So, in order to optimize the bio-MOF weight for the adsorption of the plasticizers from the aqueous medium, different weights including 5, 10, 15, 20, and 25 mg were applied. Fig. 2 illustrates that increasing NiGA MOF weight to 15 mg enhances the ERs. This phenomenon happens because increasing the bio-MOF weight provides sufficient surface area for the adsorption of the target compounds. On the other hand, increasing the weight of sorbent to 20 and 25 mg dwindles the ER values of all the analytes. The observation denotes that the use of higher than 15 mg sorbent decreases the efficiency of extraction because of agglomeration of the particles of NiGA MOF in the solution or deficient desorption of the target compounds from the bio-MOF surface. Obtaining 15 mg as the optimum weight is a blessing for the procedure since the process can be done by applying low bio-MOF weight. So, 15 mg of NiGA MOF was selected to perform the extraction process.

#### 3.2.2. Optimization of the ionic strength of DμSPE

**Fig. 3:**
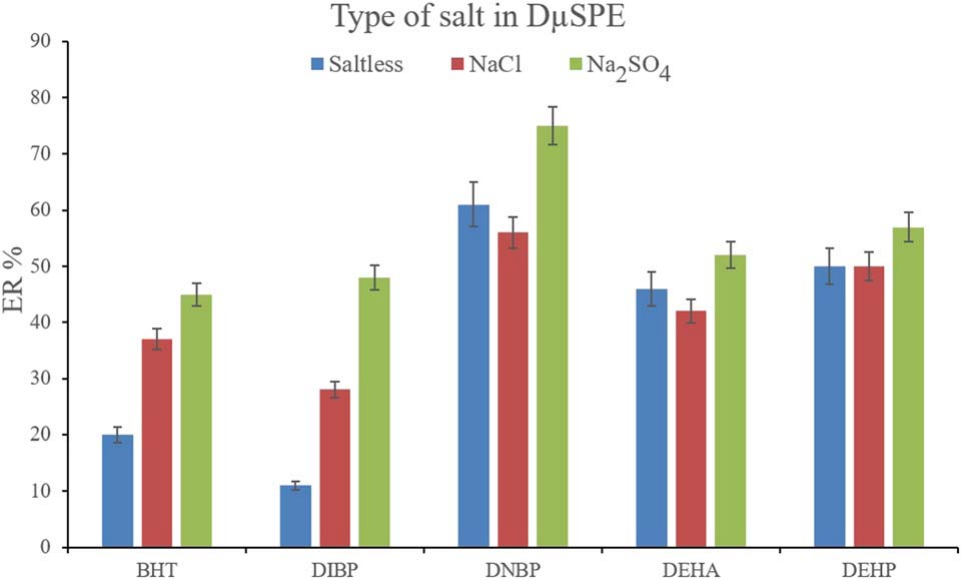
Influence of salt type on ERs of the analytes. Extraction conditions: are the same as those used in Fig. 2, except that 15 mg NiGA MOF was used.

**Fig. 4:**
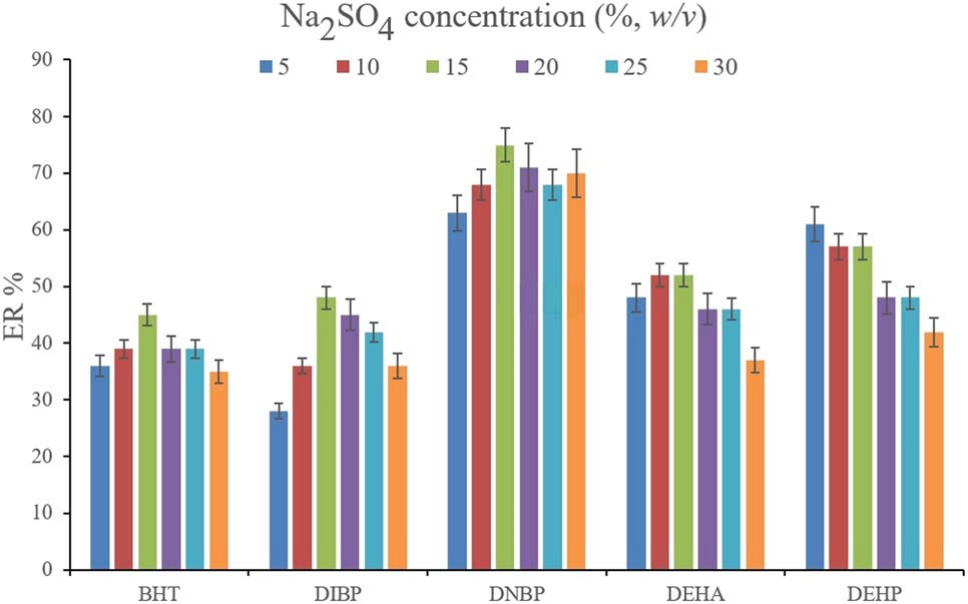
Optimization of Na_2_SO_4_ concentration. Extraction conditions: are the same as those used in Fig. 3, except that Na_2_SO_4_ was chosen as the salting-out agent.

Studying the ionic strength of a solution is of great importance to infer the efficiency of the salting-out effect on the procedure. The salting-out effect is based on reducing the solubility of the analytes in the aqueous solution in order to be extracted with higher ER values. To evaluate this effect, 15%, w/v, Na_2_SO_4_ and NaCl salts (separately) were dissolved in the aqueous solution containing the analytes and subjected to the developed extraction process. The resulted data were compared with the data obtained from the extraction of the saltless solution. Fig. 3 demonstrates the preference for Na_2_SO_4_ dissolved solution over the other tested ones. It is seen that the presence of Na_2_SO_4_ increases the ERs significantly in the case of all the target compounds. In the next step, the concentration of Na_2_SO_4_ was evaluated. For this aim, 5–30%, w/v, Na_2_SO_4_ (with intervals of 5%) were investigated. The results of the analyses are obvious in Fig. 4. It is seen that 15%, w/v, Na_2_SO_4_ enhances the ERs more than the other tested concentrations in the case of most of the analytes. It is understood that lower than 15%, w/v, Na_2_SO_4_ deficiently performs the salting-out effect so they result in lower ERs. Also, higher than the optimum concentration of Na_2_SO_4_, decreases the ERs which stems from the increased viscosity of the solution that hinders the migration of the target compounds from the aqueous solution onto the sorbent surface. So, 15%, w/v, Na_2_SO_4_ was selected.

#### 3.2.3. Optimization of vortexing time in DμSPE

**Fig. 5:**
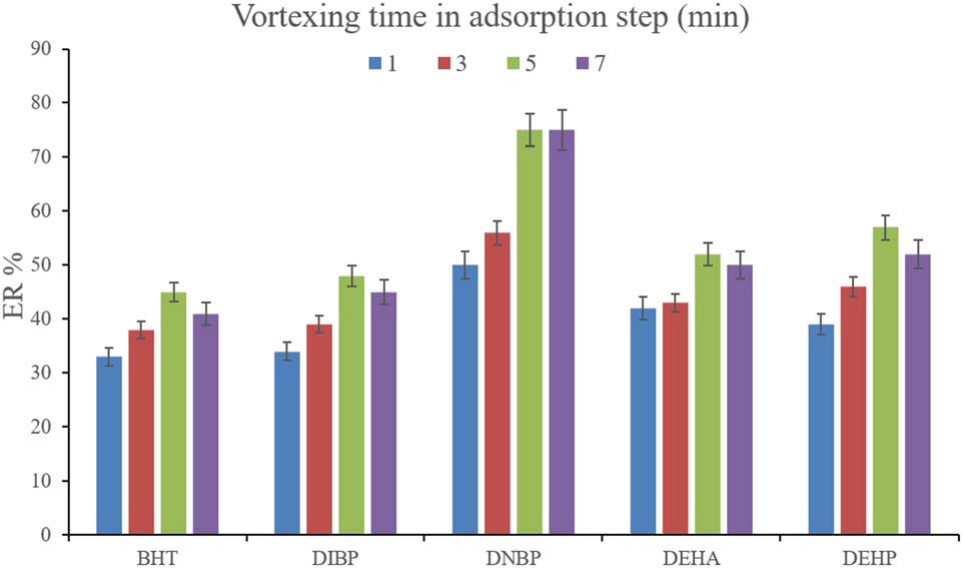
Optimization of vortexing time in the adsorption step. Extraction conditions: are the same as those used in Fig. 4, except that 15%, w/v, Na_2_SO_4_ was selected.

To facilitate the extraction of the plasticizers from the aqueous medium, vortexing can be helpful to decrease the equilibrium time. To evaluate the parameter, 1, 3, 5, and 7 min vortexing were tested and the obtained ERs are compared in Fig. 5. It is seen that 5 min vortexing is sufficient to reach the high ERs. Increasing the vortexing time to higher than 5 min has no positive consequence. Even it can lead to back extraction of the surveyed analytes which is seen by reducing the ERs of most of the analytes. So, vortexing was implemented for 5 min.

#### 3.2.4. Optimization of solution pH in DμSPE

Deviating the pH of the solution of interest in DμSPE can impact the obtained ERs. pH alteration when using bio-MOFs can significantly affect their structure and even destruct them. Also, severe basic and acidic conditions can result in the decomposition of the analytes and free O–H sections' deprotonation in NiGA MOF. The mentioned facts alter the ERs of the analytes and the solubility of the bio-MOF in the aqueous phase. The decomposition of the analytes dwindles their affinity to be adsorbed onto the bio-MOF surface. This consequence reduces their ERs. On the other hand, dissolved NiGA MOF in the solution decreases the accessible adsorption surface in the DμSPE step which leads to deficient extraction of the target compounds. This also induces lower ERs. To investigate the pH impact on the ER values of the procedure, different pH values were adjusted including 3, 5, 6, 7, 8, 9, and 10. It was seen that (data not shown here) the pH values of 8 and 7 which represent the pH of Na_2_SO_4_-dissolved solution and the neutral pH, resulted in the highest ERs. So, the process was propelled without pH alterations.

#### 3.2.5. Optimization of desorption solvent type and volume

**Fig. 6:**
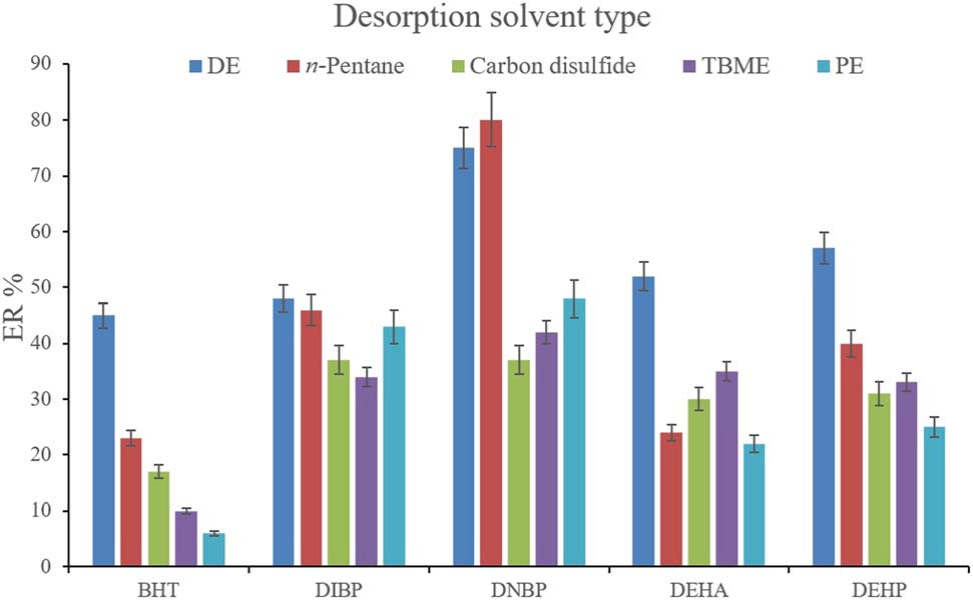
Selection of desorption solvent type. Extraction conditions: are the same as those used in Fig. 5, except that 5 min vortexing was selected.

**Fig. 7:**
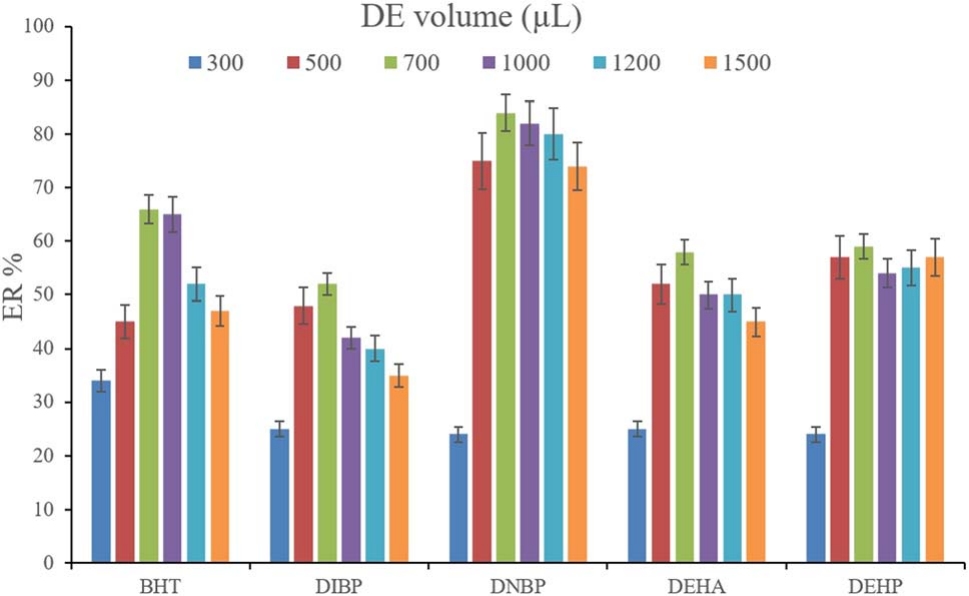
Selection of DE volume. Extraction conditions: are the same as those used in Fig. 6, except DE was used as the desorption solvent.

In order to streamline the desorption process, low boiling point organic solvents including DE, TBME, PE, *n*-pentane, and carbon disulfide (500 μL of each, separately) were applied to transfer the analytes from the NiGA MOF surface into the organic solvents. After desorption, the analyte-containing solvents were subjected to TAE under a laboratory hood. Fig. 6 demonstrates the ERs resulting from each desorption solvent. It is seen that except for the case of DNBP, DE acts as the best desorption solvent among the tested ones for the surveyed analytes. Moreover, DE has the lowest boiling point among the investigated solvents which eases its evaporation process and needs a minimum temperature to fulfill the evaporation. This is easier and more economical, and results in higher ER values. So, the volume of DE was evaluated in the next step. For this aim, 300, 500, 700, 1000, 1200, and 1500 μL of DE were used for proper desorption of the analytes. Fig. 7 shows the ERs obtained by the implementation of the mentioned DE volumes. It is seen that 700 μL application of DE results in the highest ER values for most of the analytes. Lower than 700 μL DE use leads to lower ERs that is due to inefficient desorption of the analytes due to the lack of DE volume. In the case of higher DE volumes, the ERs are also decreased. This stems from the deficiency in the subsequent dissolving of the residues. When higher than 700 μL desorption solvent is used, the solvent level in the conical bottom test tube increases. The TAE leads to the evaporation of the solvent and during the evaporation, DE leaves the tube and the analytes remain at the perimeter of it. Using 10 μL of the elution solvent cannot dissolve the analytes from higher levels of the tube *via* vortexing when using 1000, 1200, and 1500 μL of DE. But when using 700 μL of the desorption solvent, the solvent can effectively dissolve the residues at the perimeter of the tube. So, 700 μL of DE was chosen to continue the optimization steps.

#### 3.2.6. Optimization of vortexing time in the desorption step

**Fig. 8:**
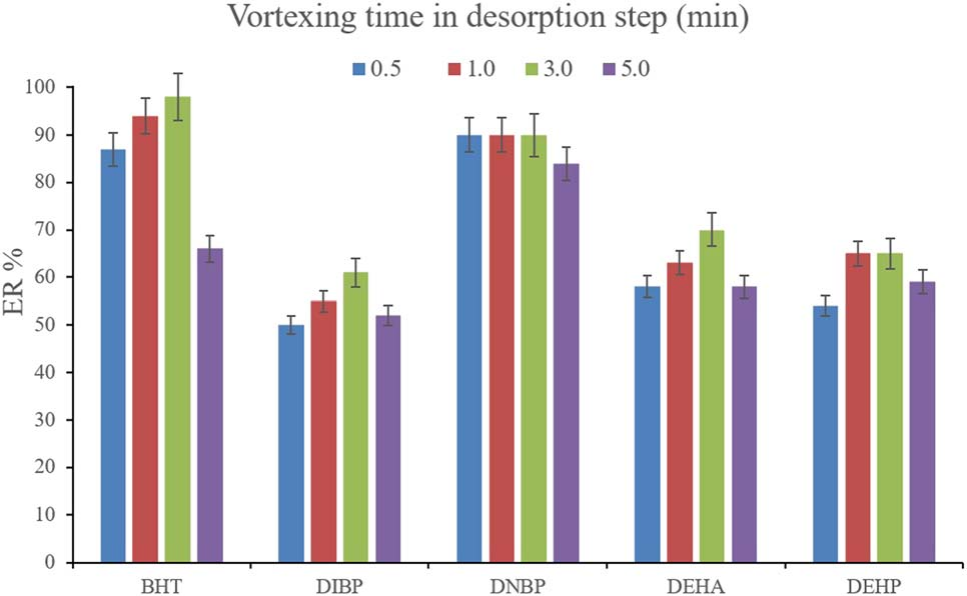
Optimization of vortexing time in the desorption step. Extraction conditions: are the same as those used in Fig. 7, except that 700 μL of DE was used.

Another parameter that determines the efficiency of desorption is vortexing time. This parameter was evaluated by the implementation of 0.5, 1.0, 3.0, and 5.0 min vortexing. The results are shown in Fig. 8. It is seen that 3 min vortexing is sufficient to reach the aim of proper desorption and increasing the vortexing time has no positive effect on the ERs. So, 3 min vortexing was implemented in the desorption step.

#### 3.2.7. Optimization of the water bath temperature

To observe the efficiency of the water bath temperature, different temperatures including 35, 55, 75, and 95 °C were set and their consequent effects on the obtained ERs were evaluated. The outcome of the experiments (data not shown here) demonstrated the priority of 35 °C over the other tested temperatures. Although increasing the bath temperature enhances the evaporation rate, it leads to analyte loss through their susceptibility to evaporation when dealing with higher temperatures. Also, maintaining the bath temperature at 35 °C is close to the boiling point of DE and it is economical and energy-saving, too. So, 35 °C temperature was set as the water bath temperature for the evaporation of the desorption solvent.

#### 3.2.8. Selection of the elution solvent type and volume

**Fig. 9:**
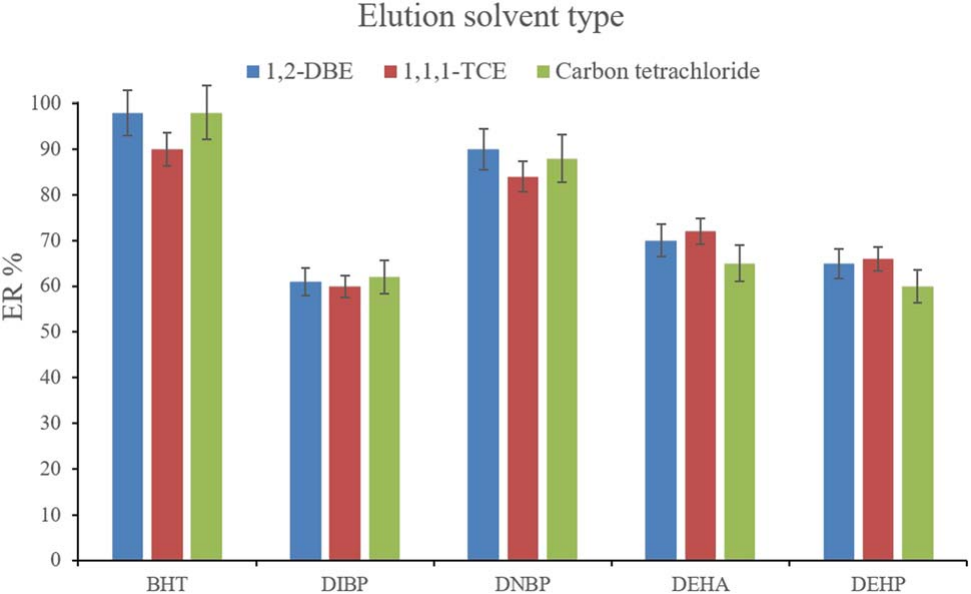
Selection of the elution solvent type. Extraction conditions: are the same as those used in Fig. 8, except that 3 min vortexing was selected for the desorption step.

After the complete evaporation of DE, 15, 10, and 15 μL of 1,1,1-TCE, 1,2-DBE, and carbon tetrachloride, respectively, were used to elute the inner perimeter of the tube containing the analytes. 10 μL of the collected phase was obtained for the tested solvents. Fig. 9 illustrates the efficiencies of the elution solvents. Although there is no significant difference among the obtained ERs for the analytes using the applied solvents, 1,2-DBE has priority over the other two solvents by resulting in higher ERs in the cases of BHT and DNBP and also less organic solvent use for dissolving the residues. So, 1,2-DBE was chosen as the elution solvent. Then, the volume of 1,2-DBE underwent evaluation by testing 10, 15, and 20 μL volumes. The obtained data (data not shown here) showed decreasing the EFs by increasing the volume of elution solvent. This stems from the dilution effect that occurs when using higher volumes. So, 10 μL of 1,2-DBE was selected.

#### 3.2.9. Optimization of vortexing time in the elution step

Vortexing the elution solvent creates a μL-level eddy of 1,2-DBE which induces the transfer of the target compounds from the inner perimeter of the test tube into the organic solvent. In order to reach the optimum conditions of vortexing in the elution step, 1, 2, 3, 4, and 5 min vortexing were implemented. The results (data not shown here) demonstrated the sufficiency of 3 min vortexing. Implementation of more than 3 min vortexing did not enhance the ERs of the analytes. So, 3 min vortexing was selected.

### 3.3. Validation of the developed method

The analytical figures of merit obtained in this study for the extraction of the analytes are presented in Table 2. Different values including linear range (LR), relative standard deviation (RSD), LOQ, LOD, coefficient of determination (*r*^2^), ER, and EF are presented in the table and discussed here. The obtained LRs were 2.64–600 μg L^−1^ for BHT, 5.74–1000 μg L^−1^ for DIBP, 3.46–1000 μg L^−1^ for DNBP, 4.72–700 μg L^−1^ for DEHA, and 5.02–500 μg L^−1^ for DEHP. The *r*^2^ values ranged from 0.993–0.998. The ER values denoting the migration of the analytes from the aqueous solution into the final organic phase were in the range of 61–98%. The EFs representing the preconcentration of the target compounds ranged from 305 to 490. According to the EFs, low LODs (0.80–1.74 μg L^−1^) and LOQs (2.64–5.74 μg L^−1^) were recorded for the method. The obtained RSDs ranged from 3.7 to 5.0% for intra- (*n* = 5) and 4.6 to 6.4% for inter-day (*n* = 3) precisions which were recorded by extracting the analytes at the concentration of 50 μg L^−1^ (of each). Appreciable ERs, high EFs, and low RSD values besides using low NiGA MOF weight for the development of the method are the highlights of the research.

**Table 2:** The obtained figures of merit for the developed method based on NiGA MOF

Analyte	LOD^a^	LOQ^b^	LR^c^	*r* ^2^ d	RSD%^e^		EF ± SD^f^	ER ± SD^g^
					Intra-day	Inter-day		
BHT	0.80	2.64	2.64–600	0.998	4.5	4.9	490 ± 5	98 ± 1
DIBP	1.74	5.74	5.74–1000	0.993	4.2	4.8	305 ± 15	61 ± 3
DNBP	1.05	3.46	3.46–1000	0.994	3.7	4.6	450 ± 10	90 ± 2
DEHA	1.43	4.72	4.72–700	0.997	4.8	6.2	350 ± 15	70 ± 3
DEHP	1.52	5.02	5.02–500	0.994	5.0	6.4	325 ± 10	65 ± 2

a Limit of detection (S/N = 3) (μg L^−1^).

b Limit of quantification (S/N = 10) (μg L^−1^).

c Linear range (μg L^−1^).

d Coefficient of determination.

e Relative standard deviation at a concentration of 50 μg L^−1^ of each analyte for intra- (*n* = 5) and inter-day (*n* = 3) precisions.

f Enrichment factor ± standard deviation (*n* = 3).

g Extraction recovery ± standard deviation (*n* = 3).

### 3.4. Analysis of real samples

**Fig. 10:**
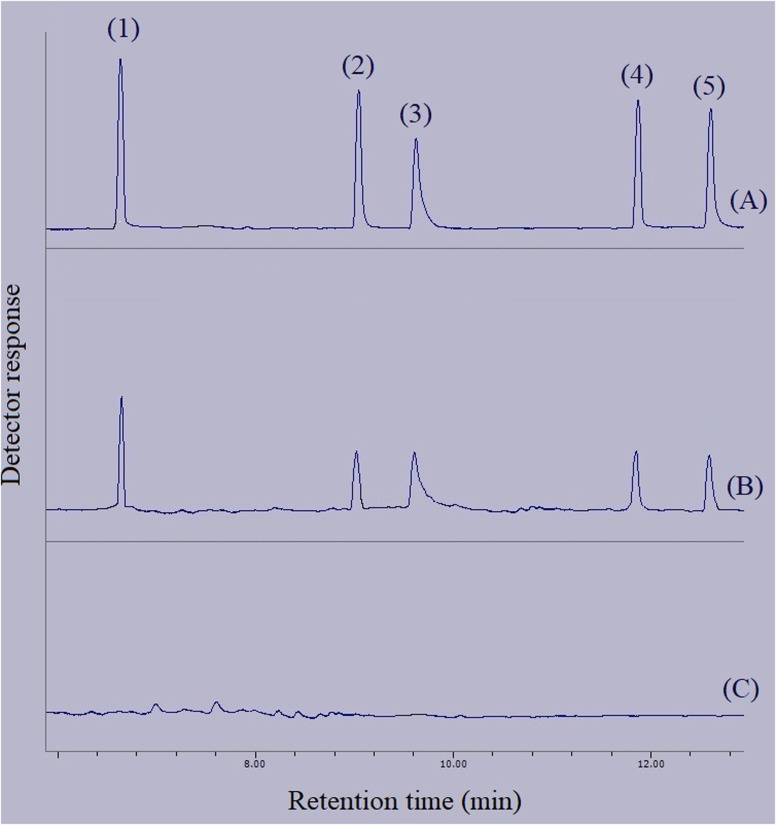
Typical GC-FID chromatograms of: (A) standard solution (250 mg L^−1^ of each analyte in methanol), (B) deionized water spiked at a concentration of 250 μg L^−1^ of each analyte, and (C) bottled water sample after performing the developed method on them, except chromatogram (A) in which direct injection without preconcentration was done. Peaks identification: (1) BHT, (2) DIBP, (3) DNBP, (4) DEHA, and (5) DEHP.

In order to connect the accomplished optimizations and drawn calibrations in the aqueous medium with the matrices of real samples, relative recovery data were calculated. Relative recovery equals the ratio of an analyte's peak area in a specific concentration extracted from the real sample to the same term extracted from deionized water multiplied by 100%. The consolidation of the relative recovery data for the extraction of the target compounds from the surveyed real samples is presented in Table 3. All the calculated relative recoveries were in the acceptable range. Fig. 10 shows three GC-FID chromatograms. They include the direct injection of 250 mg L^−1^ standard solution of the compounds of interest, extracted aqueous solution with the concentration of 250 μg L^−1^ with respect to each analyte, and the extracted bottled water sample. None of the analytes were detected in the samples.

**Table 3:** Study of matrix effect in the surveyed samples spiked at different concentrations

Analyte	Mean relative recovery ± standard deviation (*n* = 3)							
	Bottled water 1	Bottled water 2	Bottled water 3	Bottled water 4	Tap water 1	Tap water 2	Rain water 1	Rain water 2
**All samples were spiked with each analyte at a concentration of 20 μg L** ^**−**^ **^1^**								
BHT	88 ± 2	104 ± 1	91 ± 3	86 ± 3	90 ± 2	93 ± 3	107 ± 3	103 ± 1
DIBP	92 ± 2	108 ± 3	90 ± 3	90 ± 2	88 ± 3	90 ± 2	105 ± 2	107 ± 4
DNBP	95 ± 4	98 ± 3	86 ± 3	84 ± 2	89 ± 2	86 ± 3	113 ± 3	110 ± 2
DEHA	93 ± 1	95 ± 2	85 ± 4	92 ± 1	84 ± 2	94 ± 2	100 ± 2	106 ± 2
DEHP	97 ± 2	106 ± 2	84 ± 2	91 ± 2	93 ± 3	98 ± 4	104 ± 4	114 ± 3

**All samples were spiked with each analyte at a concentration of 60 μg L** ^**−**^ **^1^**								
BHT	92 ± 2	100 ± 1	87 ± 2	87 ± 2	92 ± 1	92 ± 3	103 ± 3	106 ± 4
DIBP	90 ± 3	105 ± 2	88 ± 4	93 ± 2	91 ± 3	88 ± 4	101 ± 2	110 ± 3
DNBP	96 ± 3	101 ± 4	90 ± 2	86 ± 1	94 ± 1	86 ± 4	108 ± 3	103 ± 2
DEHA	96 ± 3	96 ± 2	86 ± 3	96 ± 2	87 ± 4	90 ± 3	98 ± 2	109 ± 2
DEHP	98 ± 4	105 ± 2	88 ± 3	86 ± 2	90 ± 2	103 ± 1	109 ± 3	113 ± 1

### 3.5. Comparison of the method with similar approaches

To reveal the differences among some similar extraction procedures and also observe the priorities of the methods over each other, Table 4 consolidates the figures of merit for the developed method and the previously developed ones. The LOD and LOQ values are comparable with previous studies, except for the ones in which a mass spectrometer (MS) has been applied. MS is inherently more selective and sensitive than FID. Except for the two methods, the LRs of the study are wider than the others. The *r*^2^ values representing the linearity of the calibration curves are comparable with the given examples in the table. The RSD values are lower than most of the compared methods. Unfortunately, most of the developed methods suffer from not reporting the ER and EF values. Appreciable ERs and high EFs are also the highlights of the developed NiGA MOF-based method.

**Table 4:** Comparison of NiGA MOF-based method with some similar approaches

Method	Sample	LOD^a^	LOQ^b^	LR^c^	*r* ^2^ d	RSD^e^ (%)	EF^f^	ER^g^ (%)	Ref
DMSPE-GC-MS^h^	Water and human plasma	0.1	0.3	0.5–200	0.997	4.7–7.7	—	—	44
GDSPE-GC-MS^i^	Water samples	2	5	5–100	0.992–0.996	6-9	—	—	45
DLLME- HPLC-VWD^j^	Water samples	0.64	—	5–5000	0.999	4.3	196	—	46
HPSPE-GC-MS^k^	Water samples	0.006–0.007	0.019–0.024	0.024–10	0.991–0.999	6.7–8.1	—	—	47
SPME-GC-FID^l^	Water samples	0.032	—	1–100	0.994	4.39	—	—	48
SME-GC-MS^m^	Boiling water and pickled cucumber	0.01	0.05	0.05–100	0.996–0.999	5.9–6.2	—	—	49
HF-SPME-GC-FID^n^	Bottled drinking water	0.001–0.009	—	2–1000	0.990–0.998	6.5–9.5	930–4648	—	50
MSN-SPE-DLLME-GC-FID^o^	Water samples	0.002–0.003	—	0.01–100	0.994–0.998	4.9–7.7	17 749–21278	22–26	51
SPE-GC-MS^p^	Wine samples	100–150	166–250	250–5000	0.998–0.999	14–16	—	—	52
SPE-HPLC-UV^q^	Water samples	0.43	—	1–200	0.999	2.3	—	—	53
DμSPE-TAE-GC-FID^r^	Bottled drinking water	0.80–1.74	2.64–5.74	5.74–1000	0.993–0.998	3.7–5.0	305–490	61–98	Present method

a Limit of detection (μg L^−1^).

b Limit of quantification (μg L^−1^).

c Linear range (μg L^−1^).

d Coefficient of determination.

e Relative standard deviation.

f Enrichment factor.

g Extraction recovery.

h Dispersive magnetic solid phase extraction-gas chromatography-mass spectrometry.

i Graphene dispersive solid phase extraction-gas chromatography-mass spectrometry.

j Dispersive liquid–liquid microextraction-high performance liquid chromatography-variable wavelength detector.

k High performance solid phase extraction-gas chromatography-mass spectrometry.

l Solid phase microextraction-gas chromatography-flame ionization detection.

m Solid phase microextraction-gas chromatography-mass spectrometry.

n Hollow-fiber solid phase microextraction-gas chromatography-flame ionization detection.

o Magnetic silica nanomaterial-based solid phase extraction-dispersive liquid–liquid microextraction-gas chromatography-flame ionization detection.

p Solid phase extraction-gas chromatography-mass spectrometry.

q Solid phase extraction-high performance liquid chromatography-ultraviolet detector.

r Dispersive micro solid phase extraction-temperature assisted evaporation-gas chromatography-flame ionization detection.

### 3.6. The interaction mechanism between NiGA MOF and analytes

According to the successful extraction of the surveyed compounds from the aqueous media using the MOF, the discussion of the adsorption mechanism can be interesting. Based on the chemical structure of BHT, it can be understood that hydrogen bonds can play a significant role in the adsorption of BHT onto NiGA MOF based on the interactions between the hydrogen atom of BHT and the oxygen atoms in the MOF ligand. Moreover, π–π stacking occurs between the conjugated cyclic sections of the MOF ligand and BHT, DIBP, DNBP, and DEHP. Also, π–π stacking happens between the gallate section of NiGA MOF and the double bonds of DEHA. Nonpolar–nonpolar interactions also take place between the organic structure of the MOF and the compounds of interest. Furthermore, the organic nature of the analytes propels their adsorption onto the MOF surface based on their low solubility values in the aqueous medium. The chemical structural familiarity between the MOF ligand and BHT, DIBP, DNBP, and DEHP based on the shared cyclic section also inflames the adsorption affinity. Branched organic structures of the target compounds increase the adsorption affinity of the analytes onto the MOF structure by enhancing their organic feature. So, based on the discussed points, it can be inferred that NiGA MOF can extract the surveyed analytes from the aqueous media successfully.

## 4. Conclusions

For the first time in this study, NiGA MOF, as a green bio-MOF synthesized using nickel, gallic acid, and water was applied to develop a method based on the extraction of some plasticizers and BHT. NiGA MOF was characterized using SEM, BET, XRD, EDX, and FTIR analyses. XRD proved the crystallinity, FTIR demonstrated the creation of metal–organic bands, and EDX showed no extra elemental peak denoting the purity of the final product. TAE approach was adopted using low boiling point desorption solvents. Using DE as the desorption solvent streamlined the preconcentration by reducing the evaporation time and temperature. Bottled water, tap water, and rainwater samples were chosen to be extracted as the real samples of the study. Low bio-MOF weight (15 mg), μL-level utilization of organic solvents (700 μL of DE and 10 μL of 1,2-DBE), the greenness of the sorbent, the elimination of DLLME from the preconcentration approach, wide linear ranges (5.74–500 μg L^−1^), low LODs (0.80–1.74 μg L^−1^) and LOQs (2.64–5.74 μg L^−1^), appreciable ERs (61–98%), and high EF values (305–490) were the achievements of the developed method. In further studies, DμSPE-TAE can be adopted as a streamlined extraction and preconcentration method for the extraction of pesticides, drugs, *etc.* from different matrices due to its low cost, ease of performance, and short application time. Also, different MOFs and bio-MOFs can be tested to observe their efficiencies for the extraction of the target compounds.

## Abbreviations

DLLME: Dispersive liquid–liquid microextraction
DμSPE: Dispersive micro solid phase extraction
EF: Enrichment factor
FID: Flame ionization detector
GC: Gas chromatography
LOD: Limit of detection
LOQ: Limit of quantification
LR: Linear range
NiGA MOF: Nickel–gallic acid metal–organic framework
RSD: Relative standard deviation
TAE: Temperature-assisted evaporation

## Data availability

All data generated or analyzed during this study are included in this published article.

## Author contributions

Sakha Pezhhanfar (Bio-MOF synthesis and characterization, analytical methodology and analysis, and writing the manuscript). Mir Ali Farajzadeh (Analytical methodology and editing the manuscript). Seyed Abolfazl Hosseini-Yazdi (Synthesis methodology). Mohammad Reza Afshar Mogaddam (Analytical methodology).

## Conflicts of interest

There are no competing interests to declare.

## Supplementary Material
